# Paraherquamides – A new hope and great expectations of anthelmintic agents: Computational studies

**DOI:** 10.1371/journal.pone.0312009

**Published:** 2024-11-07

**Authors:** Anfal S. Aljahdali, Abdelsattar M. Omar, Gamal A. Mohamed, Ali M. Almalki, Sabrin R. M. Ibrahim

**Affiliations:** 1 Department of Pharmaceutical Chemistry, Faculty of Pharmacy, King Abdulaziz University, Jeddah, Saudi Arabia; 2 Department of Natural Products and Alternative Medicine, Faculty of Pharmacy, King Abdulaziz University, Jeddah, Saudi Arabia; 3 Department of Chemistry, Preparatory Year Program, Batterjee Medical College, Jeddah, Saudi Arabia; University of Mashreq, IRAQ

## Abstract

Nematode infections impose a significant health and economic burden, particularly as parasites develop resistance to existing treatments and evade host defenses. This study explores the efficacy of 48 paraherquamide analogs, a class of polycyclic spiro-oxindole alkaloids with unique structural features, as potential anthelmintic agents. Employing advanced computational methods, including molecular docking, MM-GBSA, and molecular dynamics simulations, we assessed the interaction of these analogs with the Ls-AchBP receptor, a model for nematode neurotransmission. Among the analogs studied, Paraherquamide K, Mangrovamide A, and Chrysogenamide A showed comparable docking and MM-GBSA scores to the native antagonist. Notably, their binding interactions exhibited slight distinction attributed to structural differences, such as the absence of a di-oxygenated 7-membered ring. Additionally, these analogs demonstrated robust binding stability in the molecular dynamic simulation studies and favorable pharmacokinetic properties in our in-silico ADME assessment. The insights gained from the study highlight the potential of these analogs as a basis for developing new therapeutics for nematode infections. The promising results from this computational analysis set the stage for subsequent *in-vivo* validations and pre-clinical studies, contributing to the arsenal against parasitic resistance.

## Introduction

**Fig 1:**
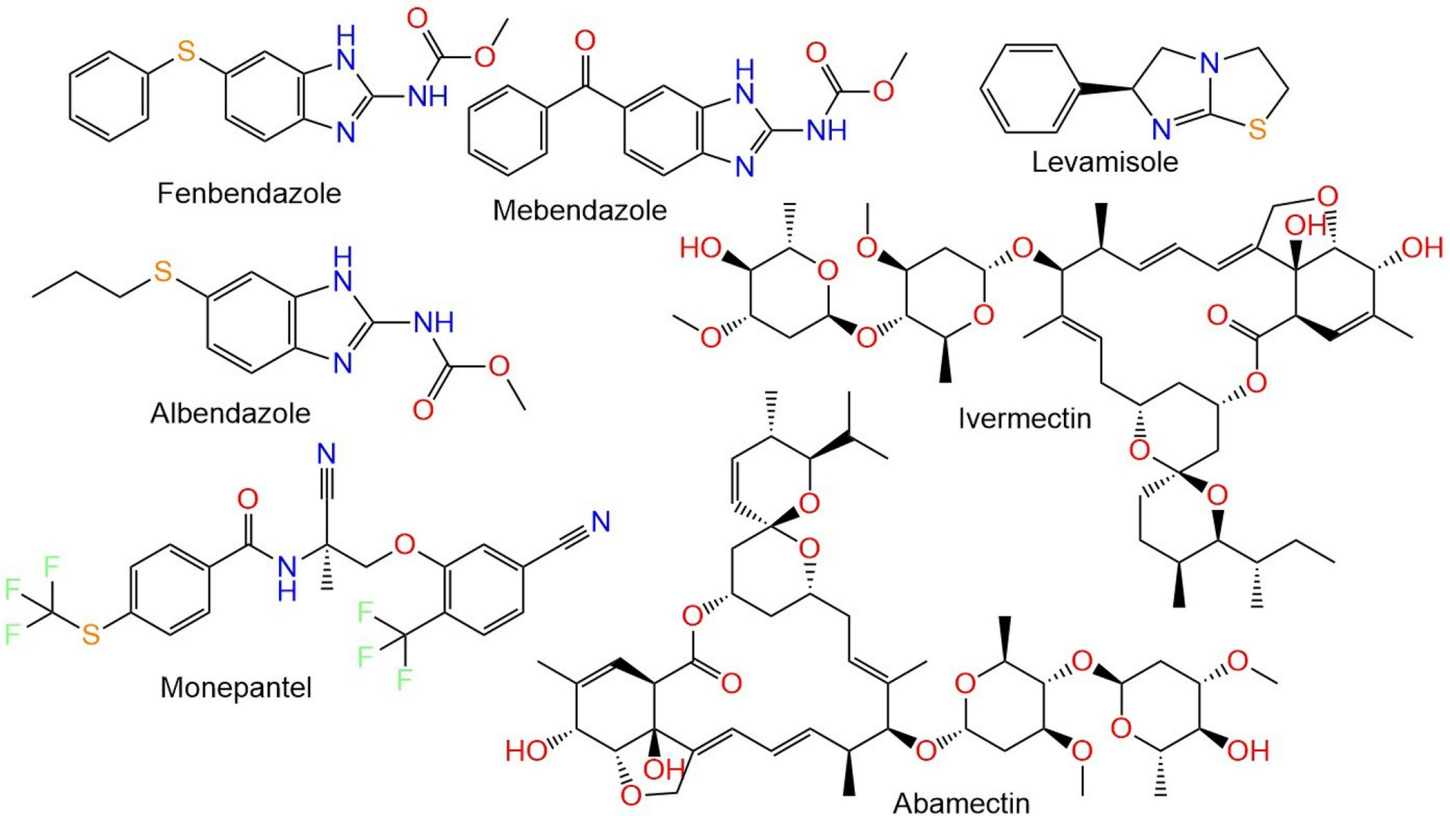
Examples of commercial anthelmintic agents.

Roundworms, also known as gastrointestinal nematodes, are a prevalent parasitic nematode encountered on humans, as well as herbivores, particularly horses and ruminants [1]. Both developing and industrialized nations are greatly impacted by parasitosis in terms of health and economy [2]. This results in decreased productivity and a decline in the overall health of small ruminant livestock with symptoms of anemia, anorexia, and diarrhea. Additionally, it causes growth retardation and affects milk, fiber, and meat production and even the animal deaths [3]. This infection is a serious health issue in subtropical and tropical regions with potential consequences, ranging from asymptomatic infection to death. Also, its existence has been linked to other human infectious diseases, including HIV and tuberculosis [3]. This infection has been effectively treated by anthelmintic medications mainly, macrocyclic lactones, benzimidazoles, and imidazothiazoles (**Fig 1**) [4].

**Fig 2:**
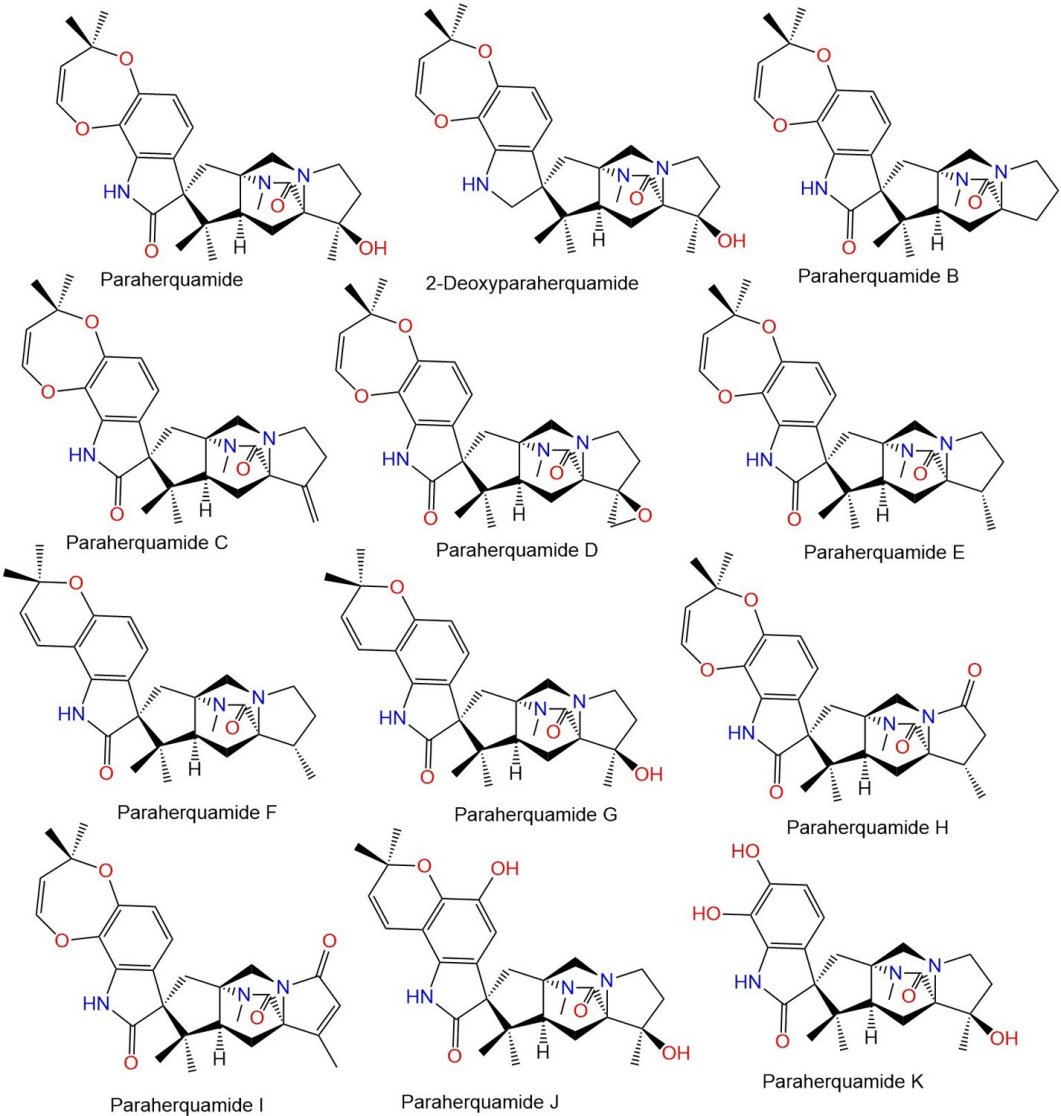
Structures of fungal paraherquamides.

**Fig 3:**
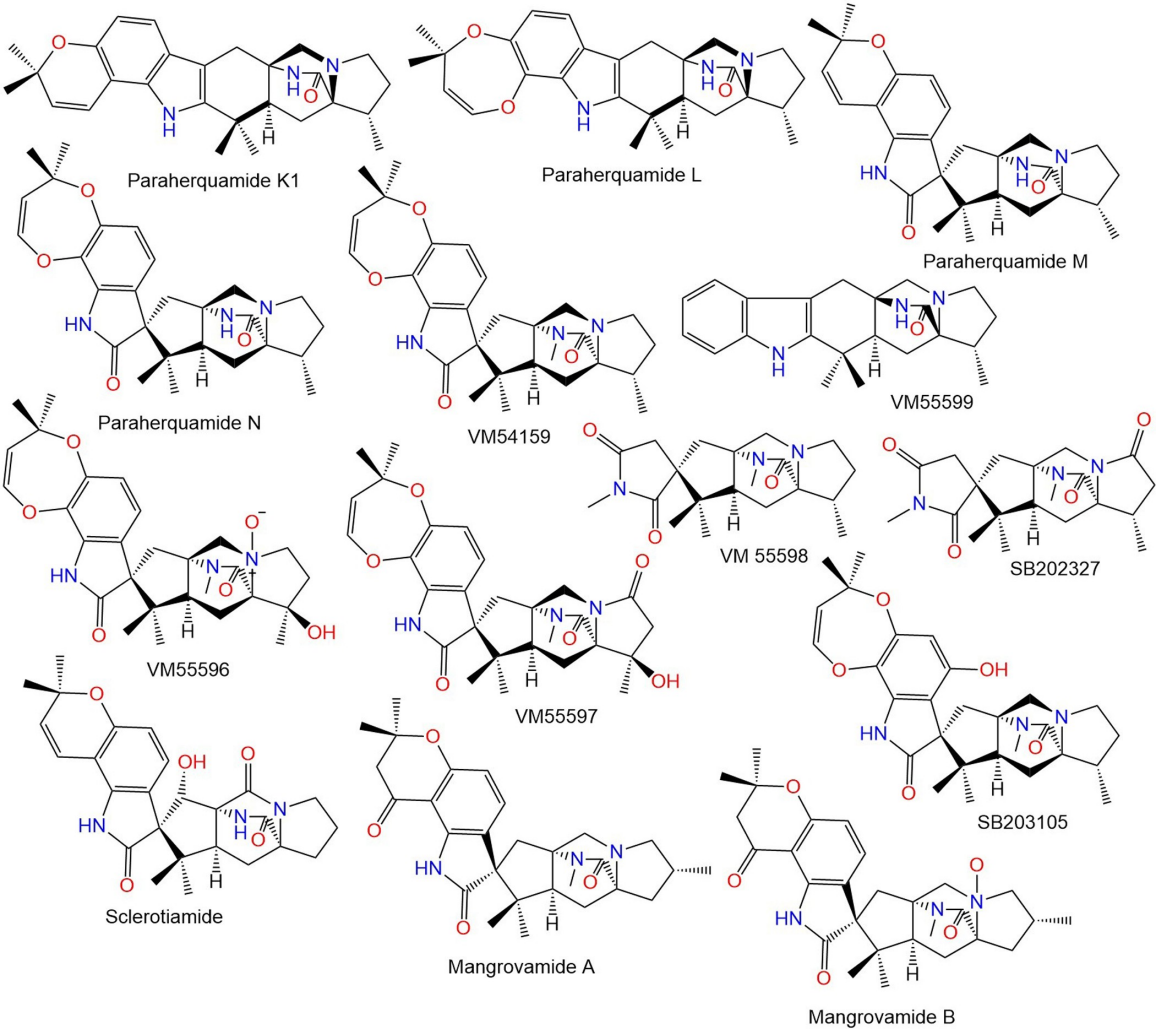
Structures of fungal paraherquamides.

**Fig 4:**
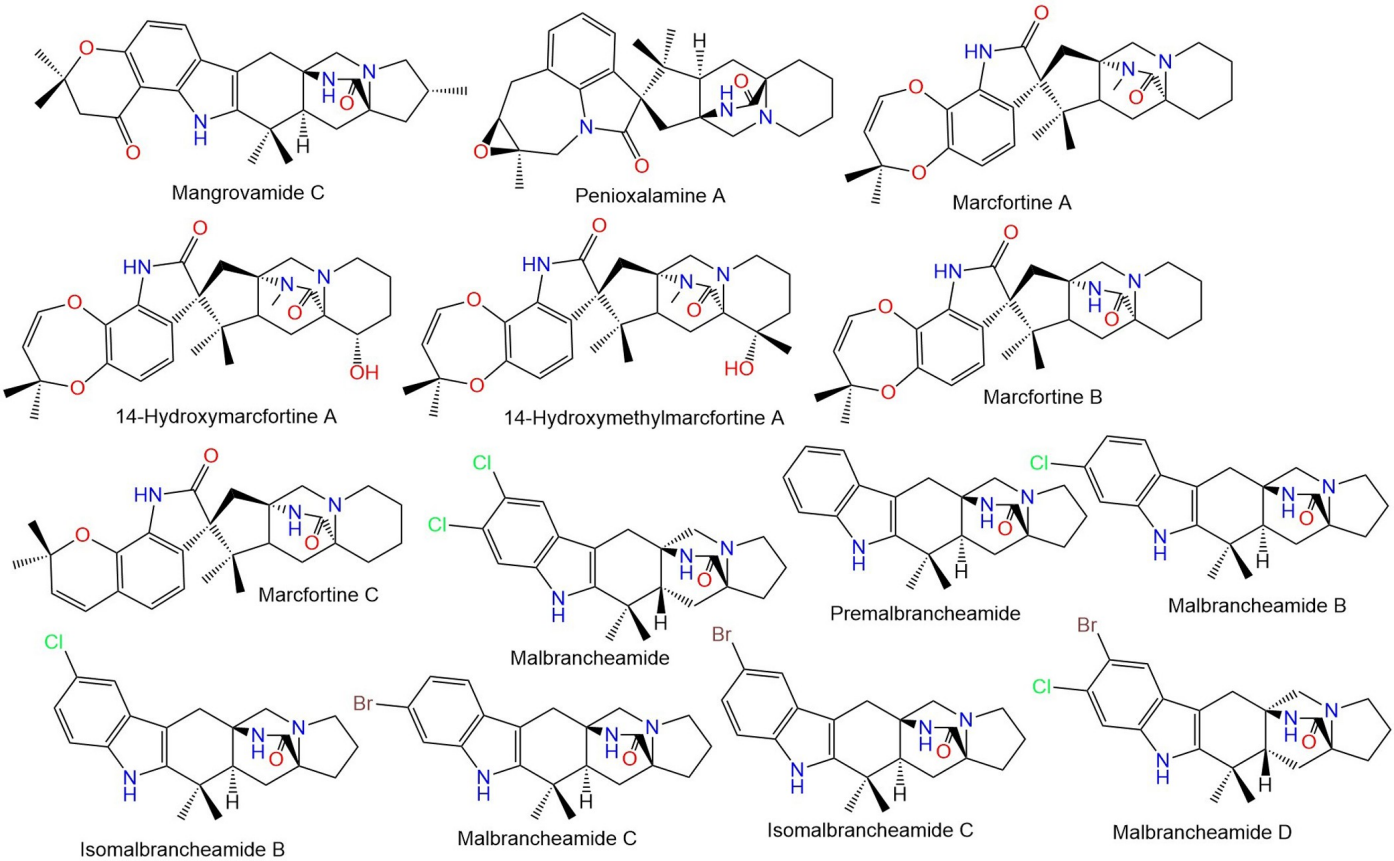
Structures of fungal paraherquamides.

**Fig 5:**
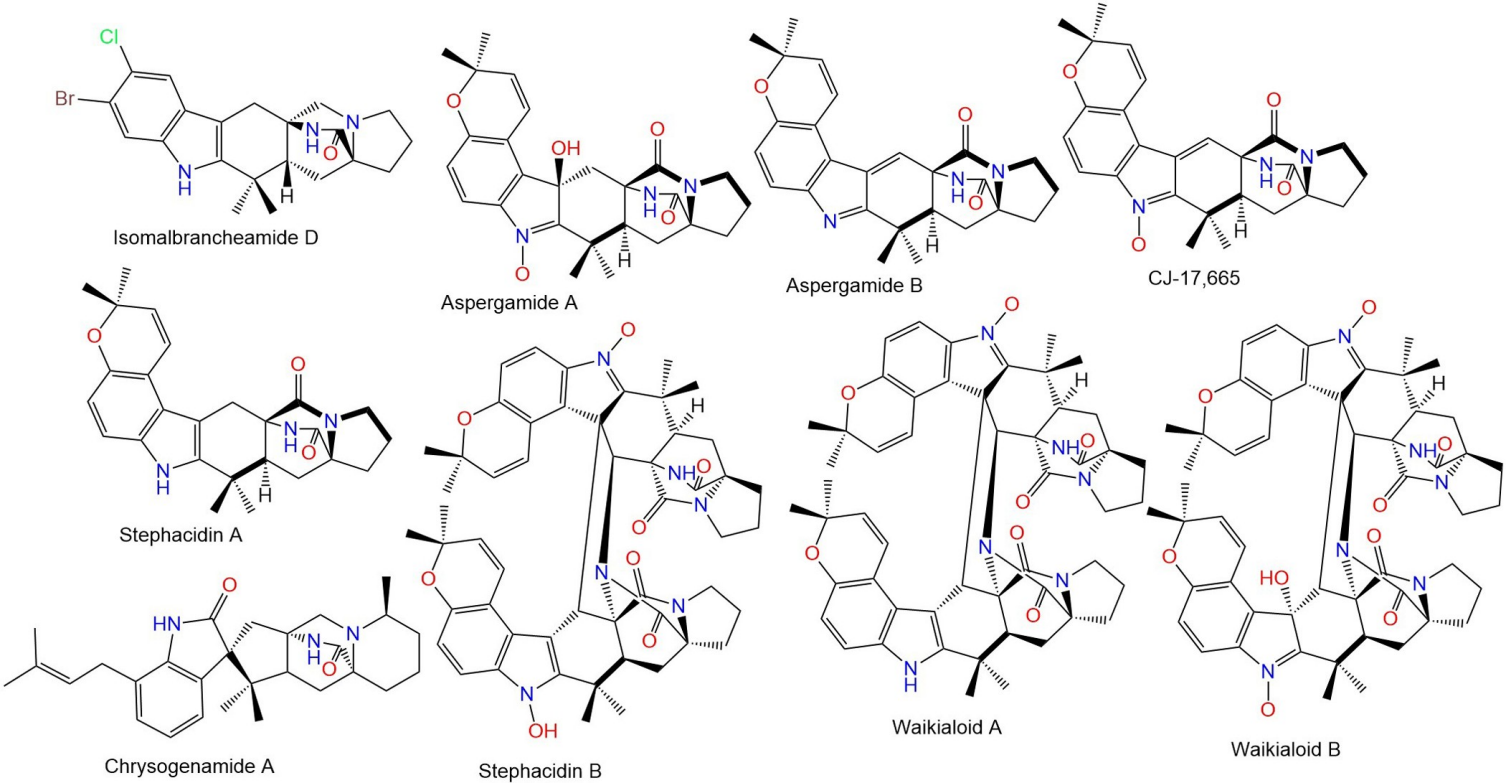
Structures of fungal paraherquamides.

The widespread misuse and use of these medications lead to developing resistance; the first occurrence of resistance was noted in sheep in 1964 [3, 5]. The last years have seen a sharp rise in anthelmintic resistance, as a result, helminth control has become a major global issue [6, 7]. Therefore, extensive efforts have been made to develop new anthelmintics or other more sustainable and effective tools for controlling nematodes [8]. The discovery of anthelmintic resistance, particularly against the widely used therapeutics, underscores an urgent need for innovative strategies and novel compounds with unique mechanisms of action to manage parasitic infections effectively. 77 of natural biomolecules, particularly those reported from plants and fungi as potential anthelmintics is currently increasing with positive outcomes [9, 10]. Paraherquamide and its related analogs are polycyclic spiro-oxindole alkaloids featuring an unusual 7-membered di-oxygenated ring connected to a tryptophan unit (**Figs 2–5)**. Besides, they contain a unique bicyclo[2.2.2]diazaoctane ring that is generated via a [4+2] intramolecular Diels- Alder reaction [11].

These metabolites are reported from various species of *Penicillium* (*P*. *paraherquei*, *P*. *janthinellum*, *P*. *chrysogenum*, *P*. *charlesii*, *P*. *simplicissimum*, *P*. *cluniae*, *P*. *oxalicum*, and *P*. *roqueforti*), *Aspergillus* (*A*. *aculeatinus*, *A*. *sclerotiorum*, and *A*. *ochraceus*), *Malbranchea* (*M*. *aurantiaca* and *M*. *graminicola*) genera (**S1 Table**). Paraherquamide was first isolated from *Penicillium paraherquei*. Earlier investigation of these metabolites, particularly paraherquamide revealed their broad-spectrum anthelmintic therapeutic potential against various parasitic worms. Ondeyka et al. stated that paraherquamide and paraherquamides B-G had antinematode potential versus *Caenorhabditis elegans* nematode genetic model, whereas paraherquamide (LD_50_ 2.5 μg/mL) was the powerful metabolite. It was noted that the dioxypino ring unsaturation and/or C-14 alkyl substitution is substantial for activity, however, C-14-OH has no role in activity [12]. In another study, paraherquamide demonstrated effectiveness (98–100%; dose 1.56 mg/kg) versus *Trichostrongylus colubriformis* infected gerbils, where it was five-folds more powerful than levamisole hydrochloride [13]. Besides, it displayed high effectiveness (≥98% reduction; dose ≥ 0.5 mg/kg) against ivermectin-resistant *Haemonchus contortus*, *Ostertagia circumcincta*, *Trichostrongylus axei*, *Trichostrongylus colubriformis (*benzimidazole- and ivermectin-resistant), and *Cooperia curticei* [14] and against *Strongyloides stercoralis* of dogs (91%) [15]. Shoop et al. revealed its marked efficacy 95% against calf’s lung and gastrointestinal nematodes (*Haemonchus placei*, *Ostertagia ostertagi*, *Cooperia oncophora*, and *Dictyocaulus viviparus*) at dose of 0.5mg/kg with no untoward effect on calves [16]. Anthelmintic testing showed that paraherquamide and its 14-de-hydroxy analog VM54159 against adult *Trichostrongylus colubriformis* L3 infections in gerbils (% fecal egg count reduction 99.5 and 100%, respectively) and *in vitro* against *Haemonchus contortus* larvae (MIC_50_s 31.2 and 15.6 μg/mL, respectively) [17, 18]. Paraherquamide and 2-deoxoparaherquamide were reported to induce their action via blocking or reversing depolarizing contractions caused by morantel and levamisole (nicotinic agonists) and acetylcholine similar to mecamylamine (nicotinic-ganglionic blocker). Also, they act as cholinergic nicotinic antagonists in both mammals and nematodes [19, 20]. Paraherquamide caused fast flaccid paralysis in the parasitic worm; *Haemonchus contortus* without altering Adenosine triphosphate (ATP) levels, suggesting a potential effect on neuromuscular receptors or the nervous system [21]. A recent study by Koizumi et al. showed that paraherquamide selectively acted on the nematode nAChRs (L-type nicotinic acetylcholine receptors). It had a higher effectiveness on the levamisole-sensitive L-type nAChR than the nicotine-sensitive N-type nAChR [22]. Interestingly, the well-known anthelmintic abamectin is utilized in conjunction with 2-deoxy-paraherquamide (derquantel) for treating gastrointestinal nematode infections in sheep [23]. Comparing the toxicity of paraherquamide and ivermectin in mice, the LD_50_s were found to be 14.9 and 29.5 mg/kg/b.wt., respectively with no observed effects at 5.6 and 18.0 mg/kg, respectively [16].

The latter studies regarding the anthelmintic potential of paraherquamide have motivated us to assess the effectiveness of other analogs (**Figs 2–5**) against nematodes L-type nAChR through in-silico approaches.

In-silico techniques, such as molecular docking and molecular dynamics (MD) simulations, have become fundamental tools in the field of drug discovery, providing valuable insights into the interactions between molecules and their biological targets [24–29]. Molecular Docking is a computational method that predicts the preferred orientation of a small molecule when bound to a protein target. This technique helps to elucidate the binding affinity and interaction mechanism of the ligand at the target site, which is essential in understanding the structure-activity relationships (SAR) crucial for the development of effective drugs [25, 30].

MD Simulation further complements docking studies by offering a dynamic view of the ligand-receptor complex over time. Unlike docking, which provides a static snapshot, MD simulations model the movements of atoms within the complex, revealing conformational changes, stability, and interaction patterns during the binding process. This dynamic analysis is crucial for understanding the long-term stability and efficacy of ligands [31, 32]. Together, molecular docking and MD simulations represent a powerful combination of tools that drive the rational design of new drugs.

Additionally, the obtained data from this investigation could guide future research efforts based on the structure-activity relationships being investigated. Therefore, paraherquamide and its analogs have emerged as potent candidates due to their broad-spectrum anthelmintic efficacy and unique mode of action, distinguishing them from the traditional anthelmintics. This distinction is crucial in the context of rapidly evolving drug resistance, as compounds with novel action mechanisms are less likely to be affected by existing resistance pathways.

Moreover, previous studies have largely focused on the bioactivity of paraherquamide without an in-depth analysis of its mechanism or the potential of its analogs. Therefore, this manuscript aims to fill a significant gap in the current understanding by employing in-silico approaches to assess the interactions of various paraherquamide analogs with the L-type nAChR of nematodes. By elucidating these interactions, our study seeks to contribute valuable insights into the structure-activity relationships of these compounds, potentially guiding the development of more effective and sustainable anthelmintic strategies. Furthermore, our research could pave the way for future investigations, focusing on the synthesis of novel analogs and their preclinical evaluation, thereby offering new avenues to combat anthelmintic resistance and enhance parasite control measures. This endeavor is not only timely but critical, given the escalating global challenge of anthelmintic resistance and the pressing need for new therapeutic options.

## Methods

### Ligand and protein preparation

The crystal structure of acetylcholine binding protein from *Lymnaea stagnalis* (*Ls*-AChBP) in complex Paraherquamide was downloaded from the protein data bank (PDB ID: 7DJI) [22]. The protein was then prepared using the “Protein Preparation Wizard” tool in Maestro software [33, 34]. This involved adding missing hydrogens to the residues, correcting the metal ionization state, and deleting water molecules greater than 5 Å from protein residues. The protein underwent refinement by predicting the pKa for the ionizable residues using PROPKA, with water molecules greater than 3 Å (not participating in water bridges) removed [35]. The protein was then energy-minimized by applying the OPLS4 force field [36]. The 48 Paraherquamide analogs in addition to Paraherquamide (**Figs 2–5**), were also prepared using the “LigPrep” tool [37]. This involved converting the 2D structures to 3D, followed by energy-minimization using the OPLS4 force field [36]. Hydrogens were added, and all possible ionization states and tautomeric forms were generated at a pH of 7.0 ± 0.2 by Epik, including a desalt option [38]. The optimization of H-bonds was carried out by predicting the pKa of ionizable groups using PROPKA [35].

### Grid generation and molecular docking

A grid box around the binding pocket of *Ls*-AChBP (PDB ID: 7DJI) was generated using Glide’s Receptor-Grid-Generation tool in the Schrödinger suite [39–41]. The box was generated around the co-crystallized ligand with default parameters, including a van der Waals radius scaling factor (1.0) and partial charge cutoff (0.25). The compounds were then docked inside the grid box using standard precision (SP) followed by an extra precision (XP) scoring function [42].

### Prime MM/GBSA calculations

Molecular mechanics with generalized born surface area (MM/GBSA) was employed to estimate the relative binding affinities for the protein ligand complex from Glide XP docking using Prime module in Schrödinger package [43–45]. The relative binding free energy ΔG_bind_ was calculated according to the following equation:

$$\Delta {\text{G}}_{\text{bind}}{=\text{E}}_{\text{complex}}\left(\text{minimized}\right)-\left[{\text{E}}_{\text{ligand}}\left(\text{unbound},\text{minimized}\right){+\text{E}}_{\text{target}}\left(\text{unbound},\text{minimized}\right)\right]$$

Where ΔG_bind_ is the predicted relative free energy for both ligand and receptor strain energy. E_complex_(minimized) denotes the MM/GBSA energy of the minimized complex, while E_ligand_ (unbound, minimized) refers to MM/GBSA energy of the ligand after separating it from the complex and allowing it to relax. E_receptor_ (unbound, minimized) represents the MM/GBSA energy of protein after removing it from the ligand. More negative values of ΔG_bind_ indicate stronger binding.

### Molecular dynamic (MD) simulations

The selected compounds, Paraherquamide K, Mangrovamide A, and Chrysogenamide A, in addition to Paraherquamide, were exported for MD simulation using Desmond/Maestro Schrödinger suite [46–48]. The system builder tool was employed to solvate the system in a TIP3P water model, and a physiological ionic concentration of 0.15 M NaCl was added to simulate the cellular environment [49]. In addition, an orthorhombic simulation box with dimensions (10 Å × 10 Å × 10 Å) around the complex was generated. The MD simulation was run using the OPLS4 force field for 100 ns at a temperature of 300 K, pH of 7.0 ± 0.2, and a standard pressure of 1.01325 bar [36]. The ensemble class was set as NPT to ensure constant temperature and pressure during the run. After completion, the results of the MD simulation were analyzed.

### ADMET properties prediction

The selected Paraherquamide analogs, Paraherquamide K, Mangrovamide A, and Chrysogenamide A, along with Paraherquamide, were subjected to in-silico ADMET prediction using the QikProp module of the Schrodinger suite [50, 51]. For each compound, various descriptors were predicted, including molecular weight (mol_MW), drug-likeness (#Stars), number of reactive functional groups present (#rtvFG), central nervous system activity (CNS), total solvent accessible surface area (SASA), number of hydrogen bond donors and acceptors (donorHB and acceptHB), predicted octanol-water partitioning (QPlogPo/w), predicted aqueous solubility (QPlogS), estimated binding to human serum albumin (QPlogKhsa), number of the possible metabolites (# metab), predicted blood-brain partitioning (QPlogBB), predicted IC_50_ for inhibiting HERG-K+ channels (QPogHERG), human oral absorption, and percentage of human oral absorption, The predicted values were then compared to the recommended range derived from values determined/observed for 95% of the known drugs.

## Results

### Molecular docking and MM-GBSA binding energy calculations

To evaluate the potential of paraherquamide analogs as anthelmintics, we employed a computational approach, docking the paraherquamide and the 48 analogs against the L-type nAChR of nematodes, specifically using the acetylcholine-binding protein model from Lymnaea stagnalis (Ls-AChBP) as a surrogate [22]. Initially, the crystallographic complex of Ls-AchBP with paraherquamide, available under PDB ID 7DJI, was utilized to guide our simulation procedures [22]. The receptor was prepared and subjected to an energy minimization process using Schrödinger`s Protein Preparation Wizard, ensuring an optimal starting conformation for interaction studies [33, 34].

**Fig 6:**
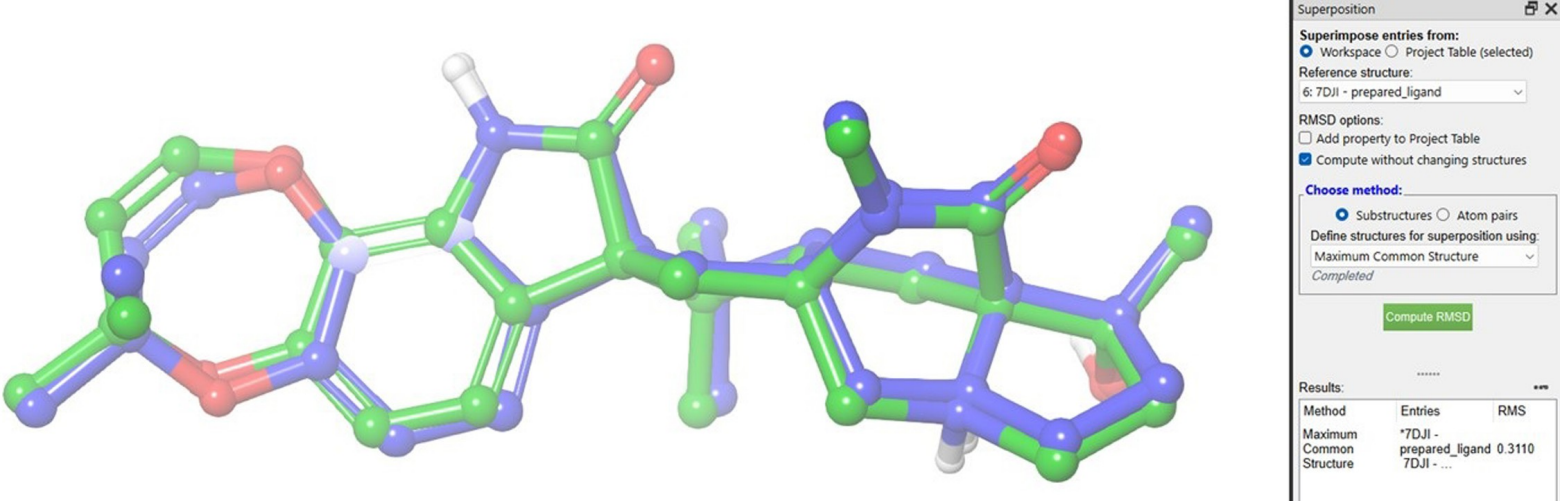
3D structure of the redocked paraherquamide superimposed on the co-crystalized paraherquamide.

Ls-AChBP, a homopentameric structure, presents five equivalent ligand-binding sites at its subunit interfaces. For computational efficiency, we focused our analysis on two adjacent subunits while omitting the remaining three. This was followed by the preparation of the paraherquamide analogs, where their two-dimensional structures were energetically optimized into three-dimensional conformations. Multiple ionization states and tautomeric forms were generated to comprehensively account for their chemical diversity [33, 37]. A targeted docking grid was generated around the known antagonist binding site using Maestros Receptor Grid Generation tool [30, 39–41]. Prior to executing the docking studies, the docking protocol`s reliability was assessed through the re-docking of the co-crystallized paraherquamide. The calculated root-mean-square deviation (RMSD) between the docked and the native antagonist conformation was determined to be 0.311 Å (**Fig 6**), substantiating the accuracy of our docking approach. The subsequent docking of the paraherquamide analogs utilized Schrödinger’s Glide software, employing both standard precision and extra precision scoring functions to refine the predictions [42].

Finally, the interaction of the complexes was evaluated using the MM-GBSA method to predict the relative binding free energies, incorporating molecular mechanics with generalized born and surface area solvation models. This analysis underscores the significance of the binding free energy, where a more negative value correlates with a greater binding affinity, thus suggesting a potential for therapeutic efficacy [43–45].

**Table 1** presents a detailed comparative analysis of docking and MM-GBSA scores for a series of Paraherquamide analogs, shedding light on their potential efficacy as anthelmintic agents. The docking analysis evaluates how each analog interacts with Ls-AChBp receptor using various scoring functions- emodel, Glide gscore, and XP gscore. The emodel scores serve as a preliminary filter, emphasizing the most energetically favorable docked positions, while the Glide gscore, based on empirical data, anticipates the likelihood of physiological binding. The XP gscore offers a more detailed assessment, focusing on precise binding affinity.

**Table 1:** Docking results and MM-GBSA scores of paraherquamide derivatives with Ls-AchBP.

Compounds	docking score	glide gscore	glide emodel	XP GScore	MM-GBSA (Kcal/mol)
**Paraherquamide K**	-17.744	-17.762	-108.872	-17.762	-109.11
**7DJI–Paraherquamide**	-17.625	-17.64	-113.012	-17.64	-116.65
**Mangrovamide A**	-16.924	-16.947	-93.993	-16.947	-106.01
**Paraherquamide N**	-16.855	-16.874	-132.284	-16.874	-107.61
**VM54159**	-16.769	-16.788	-117.987	-16.788	-112.06
**Paraherquamide E**	-16.751	-16.77	-118.021	-16.77	-111.99
**Paraherquamide G**	-16.611	-16.628	-109.218	-16.628	-116.80
**SB203105**	-16.545	-16.565	-89.35	-16.565	-98.50
**Paraherquamide B**	-16.406	-16.442	-114.41	-16.442	-108.66
**Chrysogenamide A**	-16.248	-16.354	-105.015	-16.354	-103.33
**Paraherquamide M**	-16.09	-16.109	-119.125	-16.109	-108.18

The tested compounds exhibited docking scores that are very close to that of the native antagonist, with Paraherquamide K slightly surpassing it with a gscore of -17.744 kcal/mol, marginally more negative than the native antagonist’s score of -17.625 kcal/mol. This slight yet potentially significant advantage hints at a more refined receptor interaction, which may translate to an improved anthelmintic activity. Selected for further investigation, Paraherquamide K, along with Mangrovamide A and Chrysogenamide A, stand out not only for their competitive docking scores but also for their structural divergence-most notably the absence of a di-oxygenated 7-membered ring, pointing to the possibility of unconventional receptor engagement.

The MM-GBSA scores provide a nuanced picture of binding affinity, with high negative values indicative of robust interactions. Despite being less negative than the benchmark paraherquamide, these scores still depict a strong propensity for receptor binding, underscoring their relevance in the pursuit of novel therapeutics.

**Fig 7:**
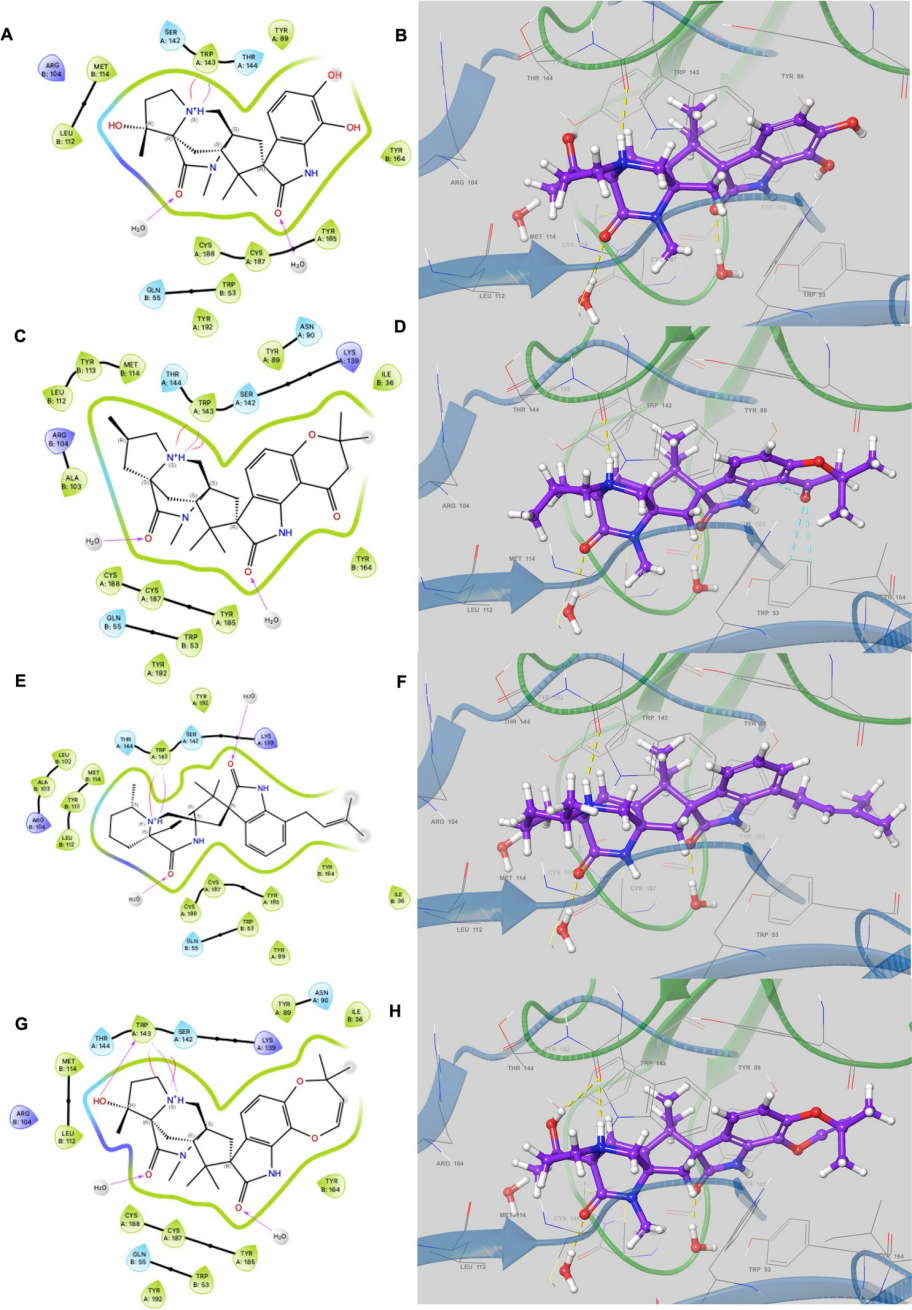
2D and 3D view of the binding interaction of *Ls*-AchBP with **(A, B)** Paraherquamide K **(C, D)** Mangrovamide A **(E, F)** Chrysogenamide A **(G, H)** Paraherquamide.

The 2D and 3D view of the selected compounds at the binding site (**Fig 7A–7F**) showed binding interactions pattern similar to that of paraherquamide (**Fig 7G and 7H**). These interactions include hydrophobic interactions with Trp53, Tyr89, Met114, Tyr185, and Tyr192 in addition to cation- π interaction with Trp143, all of which have been reported to play a critical role in the binding of paraherquamide to Ls-AchBP [22]. Additionally, they involve water-mediated H-bond interactions with the hydroxy group of Ser186 and the main chain of Cys187, as well as the OH group of Tyr164, which has been reported to determine the antagonistic activity and L-type nAChR selectivity of paraherquamide [22]. The similarity in the binding interaction of the selected paraherquamide analogs with paraherquamide suggests that the compounds exert the same biological effect.

### Molecular dynamic (MD) simulation

**Fig 8:**
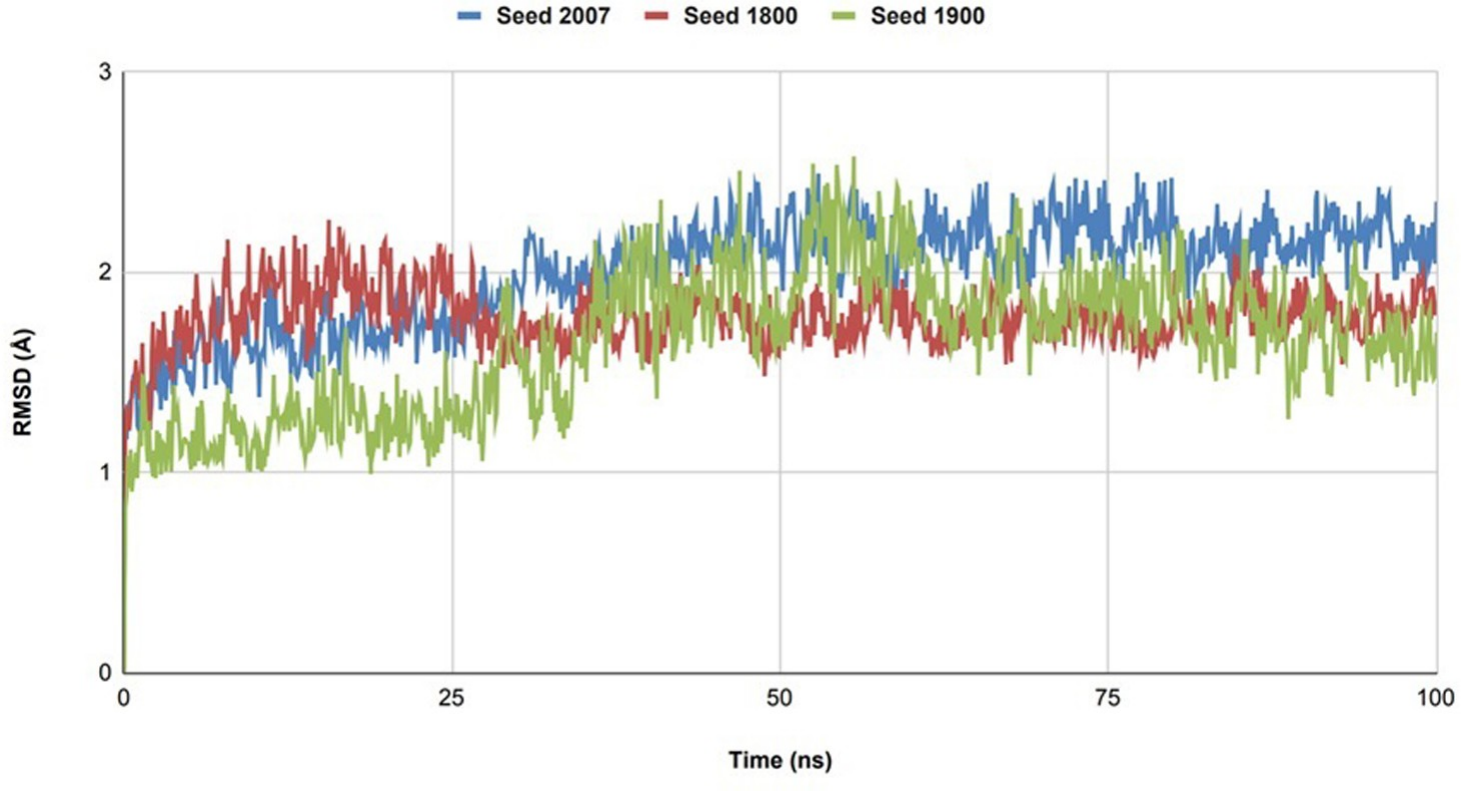
RMSD plot of the three C-alpha atoms in the paraherquamide K complex across triplicate MD simulations with different random seeds (2007, 1800, and 1900).

In our investigation into the binding dynamics of the selected Paraherquamide analogs with Ls-AchBP, MD simulations provided a pivotal platform for assessing the stability and interaction patterns of these potential anthelmintics under conditions resembling the physiological conditions. Utilizing Desmond software from the Schrödinger suite, we simulated a 100 nanoseconds timescale, carefully observing the trajectories of each analog and analyzing their interactions within the binding site through RMSD analyses and protein-ligand (PL) contact assessments [46–48]. To ensure result reliability, we performed triplicate MD simulations with different random seeds (2007, 1900, and 1800) to control initial conditions such as atomic velocities. The three C-alpha atoms of the paraherquamide K complex remained stable across all simulations, showing negligible deviation (**Fig 8**), confirming system stability.

**Fig 9:**
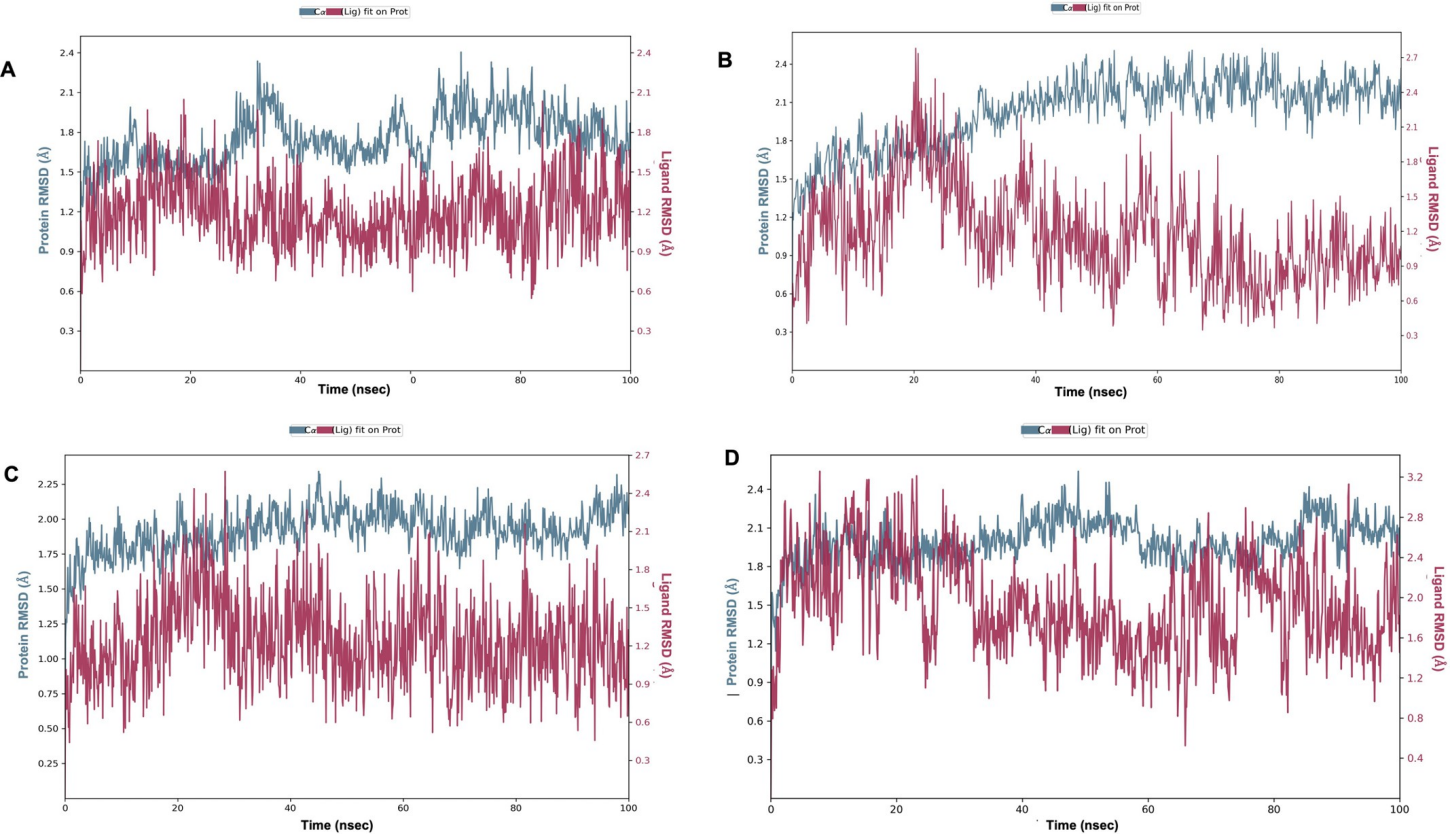
The RMSD plot for **(A)** Paraherquamide, **(B)** Paraherquamide K, **(C)** Mangrovamide A and **(D)** Chrysogenamide A.

The RMSD plots for Paraherquamide and the three analogs, Paraherquamide K, Mangrovamide A and Chrysogenamide A (**Fig 9A–9D**) indicated that the ligands maintained structural integrity within the active site, evidenced by an average deviation of 1.5 Å. Such deviations remaining within the 1 to 3 Å range exemplify stable complexes, with the ligands consistently returning to their initial conformations. This is indicative of a strong affinity and favorable binding energetics, reflecting a significant potential for these analogs to act effectively within a biological setting.

**Fig 10:**
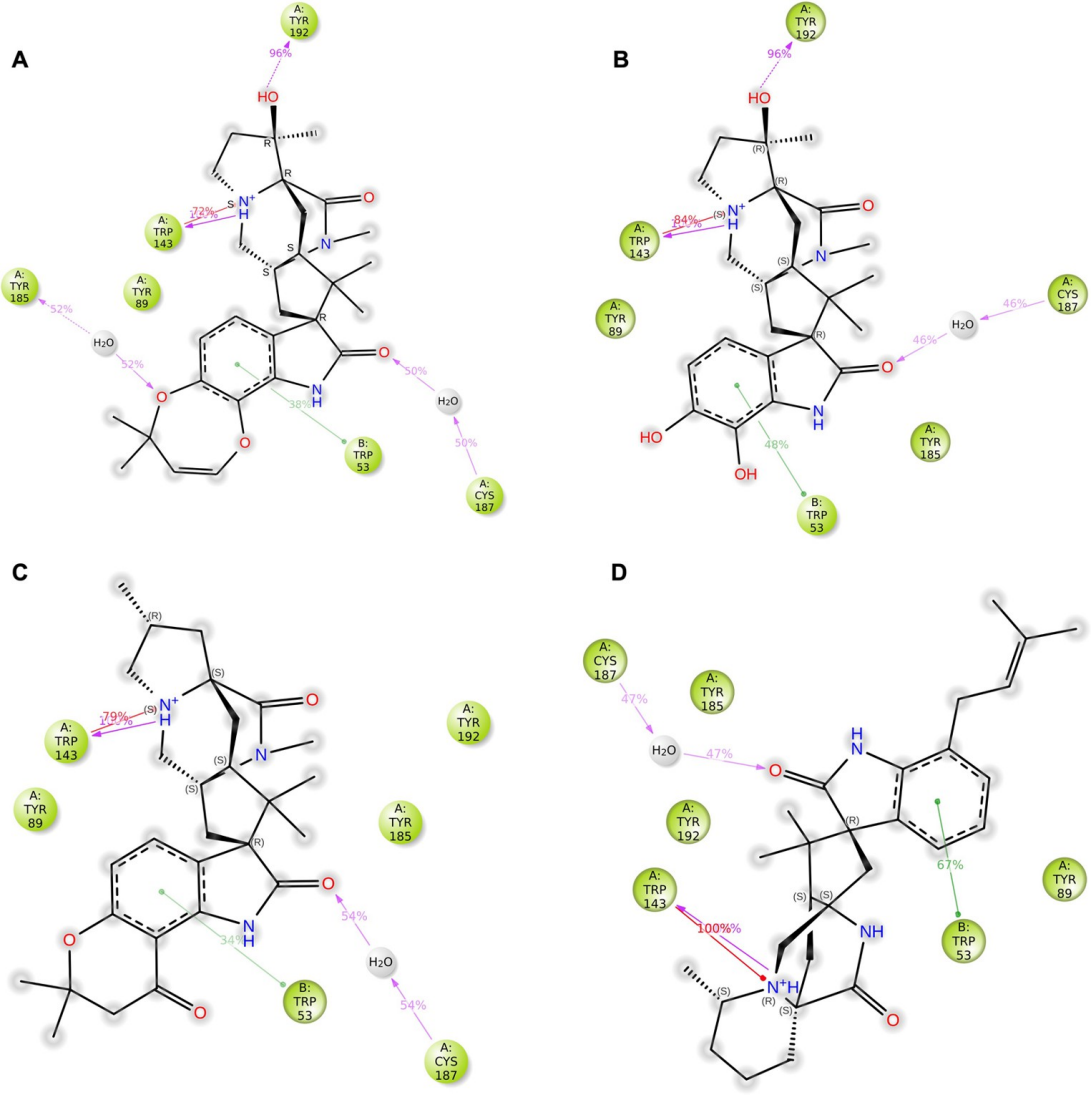
Schematic diagram showing the detailed 2D atomic interactions of *Ls*-AchBP with the **(A)** Paraherquamide **(B)** Paraherquamide K **(C)** Mangrovamide A **(D)** Chrysogenamide A. Only interactions occurred for > 30% of the simulation time in the selected trajectory (0 through 100 ns) are presented. Interactions with >100% occurrence meant that residues might have multiple interactions of a single type with the same ligand atom.

Further exploring the binding interactions, our PL contacts analysis considered only those interactions present for over 30% of the simulation time, thus highlighting the most robust and consistent interactions likely to contribute to functional activity **(Fig 10)**. Notably, both Paraherquamide and Paraherquamide K formed a persistent hydrogen bond with Tyr192, crucial for binding, present for an overwhelming 96% of the simulation. In contrast, Mangrovamide A and Chrysogenamide A, lacking the requisite hydroxyl group for hydrogen bonding, instead established hydrophobic interactions with this residue, suggesting alternative binding strategies that could circumvent resistance mechanisms.

In all the complexes, a key feature was the formation of water-mediated hydrogen bond interaction with Cys187 along with cation-π interactions involving Trp143. These interactions played a central role in firmly anchoring the compounds within the binding pocket. The ubiquitous π-π stacking interactions with Trp53 across all analogs further reinforce the hypothesis of a shared binding motif. Furthermore, we observed hydrophobic contact with Tyr185 by Paraherquamide K, Mangrovamide A, and Chrysogenamide A, while Paraherquamide engaged in water-mediated hydrogen bonding with this residue, showcasing a subtle yet potentially impactful divergence in interaction profiles.

**Fig 11:**
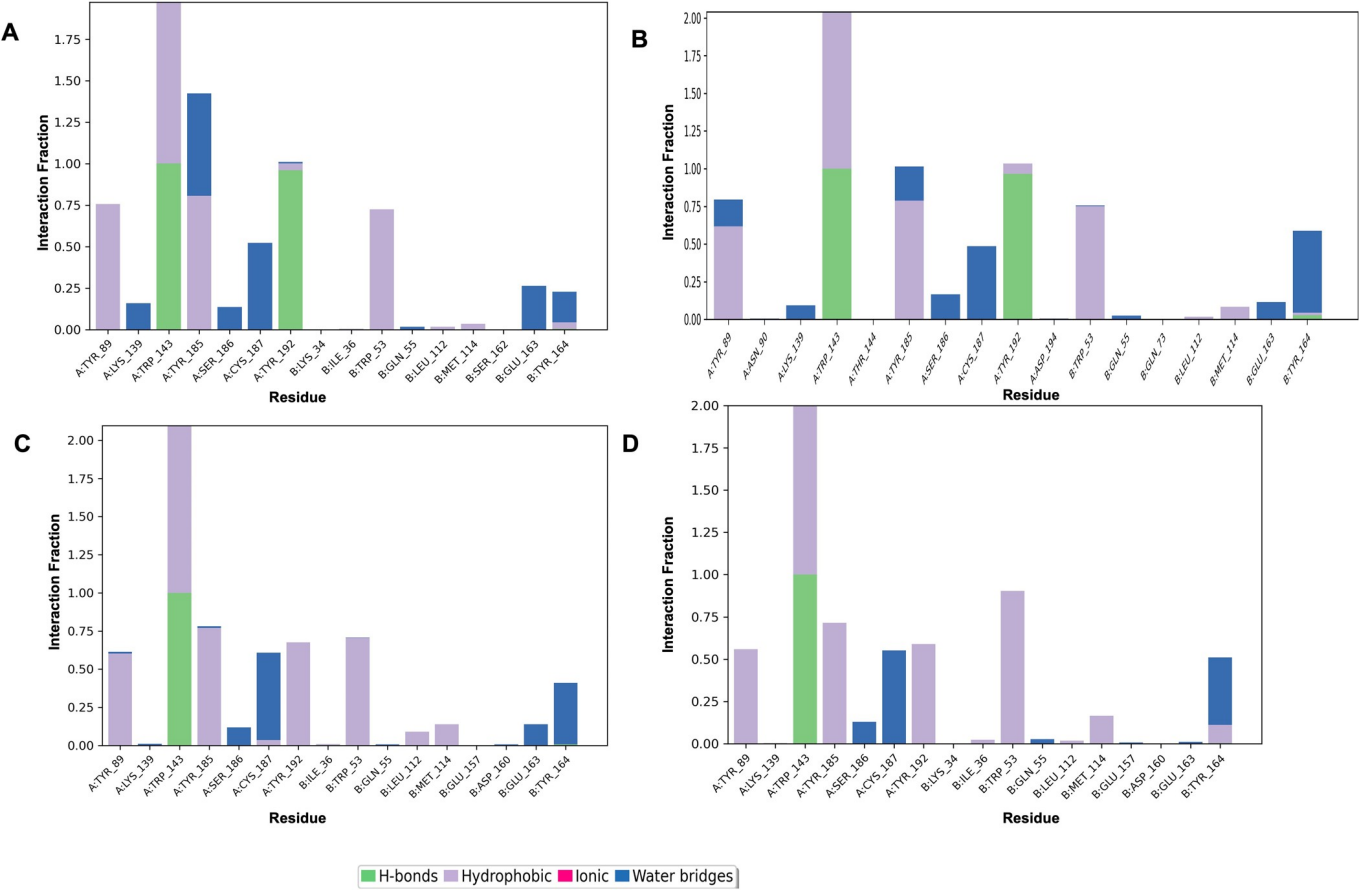
The interactions of *Ls*-AchBP with **(A)** Paraherquamide **(B)** Paraherquamide K **(C)** Mangrovamide A **(D)** Chrysogenamide A The stacked bar charts were normalized over the course of the trajectory; for example, a value of 0.8 suggested that the specific interaction was maintained during 80% of the simulation time. Values over 1.0 indicated that some protein residue might make multiple interactions of the same subtype with the ligand.

Next, a residue contacts histogram for each compound was generated (**Fig 11**). The histogram bars are color-coded to represent different interaction types, such as hydrogen bonds, hydrophobic interactions, ionic interactions, and water bridges. Comparative analysis of all the four charts showed that all the compounds exhibited the same residues contacts. The histogram showed an additional interaction that includes a water bridge interaction with Tyr164, which was not observed in the 2D scheme. This interaction fraction was slightly higher with Paraherquamide K and Chrysogenamide A in comparison to Paraherquamide.

**Fig 12:**
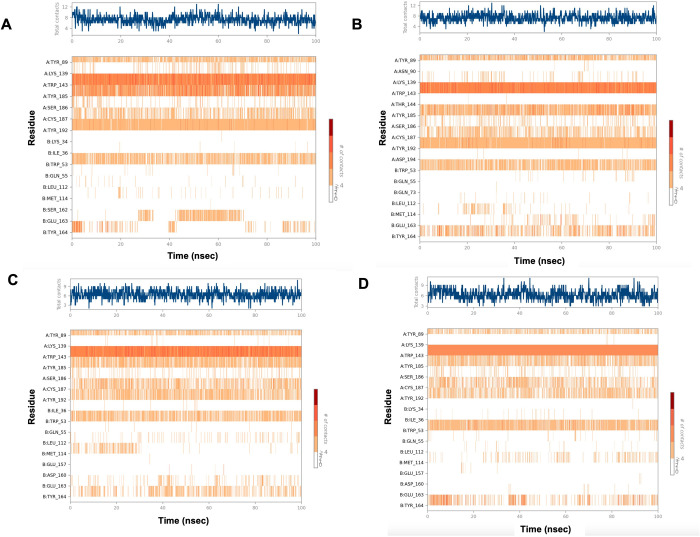
A timeline representation of *Ls*-AchBP with **(A)** Paraherquamide **(B)** Paraherquamide K **(C)** Mangrovamide A **(D)** Chrysogenamide A. The top panel depicted the total number of specific interactions of the protein with the ligand during its trajectory course. The bottom panel showed the residue interactions with the ligand in each trajectory frame. The dark orange color indicated the presence of more than one interaction between some residues and the ligand.

**Fig 12** illustrated the timeline representation of PL interactions for the four complexes. The top panel displayed the number of specific contacts the protein makes with the ligand over the course of the MD trajectory. The bottom panel showed the residues that interact with the ligand in each trajectory frame. A consistent dark orange color in the bottom panel indicates the involvement of residue in several interactions with the compound throughout the trajectory. From the chart generated for each complex **(Fig 12)**, it was observed that several residue contacts were common among all compounds. These residues include Tyr89, Trp143, Tyr185, cys187, Tyr192, and Trp53. Additionally, the interaction with Tyr164 was observed to be more persistent throughout the simulation period with Paraherquamide K, Mangrovamide A, and Chrysogenamide A compared to the native antagonist, Paraherquamide.

### In-silico ADMET properties of selected ligand

The selected paraherquamide analogs, Paraherquamide K, Mangrovamide A, and Chrysogenamide A, underwent a comprehensive assessment of their drug-likeness and ADMET (absorption, distribution, metabolism, excretion, and toxicity) properties using the QikProp module within Schrodinger’s Maestro software [50, 51]. QikProp utilized the 3D structures of the compounds to predict their pharmacokinetics and physiochemical properties, comparing them with those of 95% of known drugs. This predictive analysis helped in identifying compounds that might pose challenges during clinical phases of drug discovery and development, thereby reducing the likelihood of failures in the process.

Key descriptors generated by QikProp include various factors, such as stars, number of reactive functional groups (rtvFG), predicted central nervous system activity (CNS), molecular weight (mol_MW**)**, total solvent accessible surface area (SASA), total solvent accessible volume (volume), estimated number of hydrogen bonds donor and acceptor (donorHB and acceptHB), water/gas and octanol/water partition coefficients (QPlogPw and QPlogPo/w), predicted aqueous solubility (QplogS), Predicted IC_50_ value for blockage of HERG K+ channels (QPlogHERG), apparent Caco-2 cell permeability (QPPCaco), predicted brain/blood partition coefficient (QPlogBB), number of likely metabolic reactions (#metab‡), prediction of binding to human serum albumin (QPlogKhsa), human oral absorption, and % oral absorption values.

Notably, all descriptors for the compounds, except for Paraherquamide K, fell within the acceptable range. Paraherquamide K exhibited poor QPPCaco and oral absorption, which can be attributed to its polarity. As shown in **Table 2**, the compounds exhibited favorable physicochemical properties that suggested a strong candidacy for oral administration, a key consideration in drug development.

**Table 2:** In-silico ADME (absorption, distribution, metabolism, and excretion) properties analysis of the selected compounds using via QikProp.

Molecule	Recommended range	Paraherquamide	Paraherquamide K	Mangrovamide A	Chrysogenamide A
**#stars**	0–5	0	0	0	0
**#rtvFG**	0–2	0	0	0	0
**CNS**	−2 (inactive) +2 (active)	1	**−2**	1	1
**mol_MW**	130.0–725.0	493.602	427.499	477.602	447.619
**SASA**	300.0–1000.0	719.304	630.753	753.265	726.724
**volume**	500.0–2000.0	1406.862	1207.598	1435.133	1399.753
**donorHB**	0.0–6.0	1	3	0	2
**accptHB**	2.0–20.0	7.5	7.75	8.25	6
**QPlogPw**	4.0–45.0	12.836	16.064	12.731	13.313
**QPlogPo/w**	−2.0–6.5	3.214	0.915	2.637	3.904
**QPlogS**	−6.5–0.5	-4.495	-2.638	-4.171	-4.658
**QPlogHERG**	concern below −5	-3.776	-3.336	-4.141	-4.09
**QPPCaco**	<25 poor, > 500 great	181.267	**16.863**	87.127	301.553
**QPlogBB**	−3.0–1.2	-0.055	-1.079	-0.371	0.161
**#metab**	1–8	13	3	1	3
**QPlogKhsa**	−1.5–1.5	0.599	-0.03	0.256	0.807
**Human Oral Absorption**	1, 2, or 3 for low, medium, or high	3	2	3	3
**Percent Human Oral Absorption**	>80% is high	86.184	**54.262**	77.109	82.585

The analysis returned zero stars (#stars) for all compounds, indicating adherence to Lipinski’s rule of five and the absence of reactive or toxic functional groups, which boded well for their oral bioavailability. Additionally, the lack of reactive toxic functional groups (#rtvFG) further supported their suitability as drug candidates.

The CNS scores for the compounds suggested varying levels of central nervous system activity. Paraherquamide K`s score of −2 implied it would likely be inactive in the CNS, potentially reducing the risk of CNS-related side effects, which is particularly advantageous for drugs intended for systemic infections. The scores of 1 for Paraherquamide, Mangrovamide A, and Chrysogenamide A may indicate moderate CNS penetration, which could be explored for therapeutic benefit if CNS involvement is desired.

Molecular weights within the optimal range indicate a likely favorable pharmacokinetic profile, with no molecule exceeding the upper limit, which can be associated with poor absorption and permeability. The solvent accessible surface area (SASA) and volume measurements lie within the ideal parameters, suggesting that these molecules may interact effectively with their biological targets without being hindered by size or steric bulk.

Hydrogen bond donors and acceptors are in line with the recommended values, suggesting a balance that may contribute positively to both solubility and permeability, factors that are critical for oral absorption and interaction with biological targets. In particular, the higher number of donor hydrogens in Paraherquamide K could influence its solubility and receptor binding profile.

Partition coefficients, which are indicative of the lipophilicity of the compounds, fall within the optimal range, suggesting they possess appropriate lipophilic properties to passively diffuse through biological membranes, without being excessively lipophilic, which could lead to solubility issues or nonspecific binding.

The water solubility (QPlogS) predictions for all compounds, while negative, are not excessively so, hinting at a solubility that is likely to be conducive to adequate absorption. The hERG inhibition risk is a crucial safety parameter, and the compounds’ scores above the concerning threshold suggest a lower risk for cardiotoxicity, a vital consideration for safety profiles.

Caco-2 permeability predictions are encouraging, indicating these compounds are expected to be permeable enough to be absorbed intestinally. Varying degrees of predicted blood-brain barrier permeability (QPlogBB) could open avenues for therapeutic targeting within the CNS if such an action is desired.

Metabolic stability, as indicated by the number of predicted metabolites (#metab), appears varied, with Paraherquamide potentially undergoing more extensive metabolism, which could impact its efficacy and duration of action. The plasma protein binding predictions (QPlogKhsa) fall within the optimal range, suggesting a balanced duration of action and free drug concentration for activity.

The human oral absorption scores, in conjunction with the percent human oral absorption predictions, are highly encouraging. High scores across most compounds suggest that these analogs are likely to be well-absorbed when administered orally, which is essential for patient compliance and treatment efficacy.

Overall, the in-silico ADME analysis indicates that the Paraherquamide analogs have a profile that suggests a strong potential for development into orally active anthelmintic medications.

## Discussion

In this study, we aimed to assess the potential paraherquamide analogs as anthelmintic treatment using computational techniques, including molecular docking, MM-GBSA scoring and molecular dynamics simulations. Our goal was to identify s with strong binding affinity to the L-type nAChR receptor, lying the groundwork for further experimental validation.

Our MM-GBSA scores revealed notable binding affinities for several paraherquamide analogs, albeit slightly less favorable than the benchmark paraherquamide. The docking analysis using multiple scoring functions further emphasized the potential of these analogs to interact with the L-type nAChR receptor, suggesting strong physiological binding characteristics.

As we progressed, molecular dynamics (MD) simulations played a crucial role in validating our docking results by exploring the dynamic nature of ligand-receptor interactions. These simulations provided insights into stability and conformational evolution that static docking cannot capture. MD simulations allowed us to explore the flexibility of the binding pocket, strength of ligand-receptor bonds over time, and implications of these dynamic interactions for therapeutic effectiveness.

Our findings also underscored the need for in-depth pharmacokinetic investigations due to observed variations in CNS activity, metabolic stability, and plasma protein binding profiles across the analogs. Addressing these factors will be critical for translating computational predictions into effective therapeutic strategies.

Moving forward, subsequent in-vitro and in-vivo studies are warranted to confirm the in-silico predictions and fully elucidate the pharmacological profiles of these analogs. This discussion lays the foundation for advancing these promising candidates towards the development of novel anthelmintic treatments, driving us closer to the potential realization of these analogs as new entries in the anthelmintic drug arsenal.

## Conclusions

Paraherquamide-type molecules belong to the indole alkaloids family that are commonly biosynthesized by various *Penicillium* species. The integrated computational and in-silico study has shed light on the promise held by Paraherquamide analogs as potential anthelmintic agents. Through a combination of docking studies, molecular dynamics simulations, and ADME profiling, we have characterized these compounds’ binding efficacies, dynamic stabilities, and pharmacokinetic properties, painting a compelling picture of their therapeutic viability.

The consistent binding stability observed in the MD simulations, particularly the key interactions with residues such as Tyr192 and Trp143, along with favorable ADME profiles, underscores the potential for oral efficacy. Collectively, the extensive MD simulations have provided a deep understanding of the interaction landscape of Paraherquamide analogs within Ls-AchBP, highlighting stability and consistent interaction patterns that are promising for anthelmintic efficacy. The diversity in interaction profiles among the analogs opens avenues for the tailored design of compounds to overcome resistance issues common in current anthelmintic treatments.

## Supporting information

S1 Table Naturally occurring paraherquamides and their analogues (Fungal source, host, place, molecular weights, and formulae). (DOCX)
