# Supplementary material for: Paraherquamides – A new hope and great expectations of anthelmintic agents: Computational studies
Source: PLoS One. 2024 Nov 7;19(11):e0312009. doi: 10.1371/journal.pone.0312009 (PMC11542861; doi:10.1371/journal.pone.0312009)
Supplement: S1 Table — (DOCX) [file pone.0312009.s001.docx]

**Table S1**

Naturally occurring brevianamides, paraherquamides and their analogues (Fungal source, host, place, molecular weights, and formulae).

| **Compound name** | **Fungus** | **Source, Place** | Ref. |
| --- | --- | --- | --- |
| Paraherquamide = VM29919 = PNU-97333 | *Penicillium paraherquei* | Cultured | [1-3] |
|  | *Penicillium janthinellum* HK1-6 | Mangrove rhizosphere soil-derived | [4] |
| 2-Deoxyparaherquamide = PNU-141962 | *-* | - | [3] |
| Paraherquamide B | *Penicillium charlesii* ATCC 20841 | Cultured | [2,5] |
| Paraherquamide C | *Penicillium charlesii* ATCC 20841 | Cultured | [2,5] |
| Paraherquamide D | *Penicillium charlesii* ATCC 20841 | Cultured | [2,5] |
| Paraherquamide E | *Penicillium charlesii* ATCC 20841 | Cultured | [2,5] |
|  | *Penicillium janthinellum* HK1-6 | Mangrove rhizosphere soil-derived | [4] |
|  | *Aspergillus aculeatinus* WHF0198 | Sediments, deep-sea of the South China Sea | [6] |
| Paraherquamide F = VM55594 | *Penicillium charlesii* ATCC 20841 | Cultured | [2,5] |
|  | *Penicillium* sp. | Cultured | [7] |
| Paraherquamide G = VM54158 | *Penicillium charlesii* ATCC 20841 | Cultured | [2,5] |
|  | *Penicillium* sp. | Cultured | [7] |
|  | *Penicillium simplicissimum* ATCC 20841 | Cultured | [8] |
| Paraherquamide H | *Penicillium cluniae* Quintanilla CECT 2888 | Cultured | [9] |
| Paraherquamide I | *Penicillium cluniae* Quintanilla CECT 2888 | Cultured | [9] |
| Paraherquamide J | *Penicillium janthinellum* HK1-6 | Mangrove rhizosphere soil-derived | [4] |
| Paraherquamide K* | *Penicillium janthinellum* HK1-6 | Mangrove rhizosphere soil-derived | [4] |
| Paraherquamide K* | *Penicillium simplicissimum* ATCC 20841 | Cultured | [8] |
|  | *Aspergillus aculeatinus* WHF0198 | Sediments, deep-sea of the South China Sea | [6] |
| Paraherquamide L | *Penicillium simplicissimum* ATCC 20841 | Cultured | [8] |
| Paraherquamide M = VM55595 | *Penicillium* sp. IMI 332995 | Cultured | [10] |
|  | *Penicillium simplicissimum* ATCC 20841 | Cultured | [8] |
|  | *Aspergillus aculeatinus* WHF0198 | Sediments, deep-sea of the South China Sea | [6] |
| Paraherquamide N = SB200437 | *Penicillium* sp. IMI 337664 | Cultured | [11] |
|  | *Penicillium janthinellum* HK1-6 | Mangrove rhizosphere soil-derived | [4] |
|  | *Penicillium simplicissimum* ATCC 20841 | Cultured | [8] |
|  | *Aspergillus aculeatinus* WHF0198 | Sediments, deep-sea of the South China Sea | [6] |
| VM54159 | *Penicillium* sp. | Cultured | [7,11] |
| VM55599 | *Penicillium* sp. IMI 332995 | Cultured | [10] |
|  | *Aspergillus aculeatinus* WHF0198 | Sediments, deep-sea of the South China Sea | [6] |
| VM55596 = N-oxide paraherquamide A | *Penicillium* sp. IMI 332995 | Cultured | [10] |
| VM 5597 | *Penicillium* sp. IMI 332995 | Cultured | [10] |
| VM55598 = Aspergillimide | *Penicillium* sp. IMI 337664 | Cultured | [11] |
| SB202327 = 16-Keto-aspergillimide | *Penicillium* sp. IMI 337664 | Cultured | [11] |
| SB203105 | *Penicillium* sp. IMI 337664 | Cultured | [11] |
| Aculeaquamide A | *Aspergillus aculeatinus* WHF0198 | Sediments, deep-sea of the South China Sea | [6] |
| Sclerotiamide | *Aspergillus sclerotiorum* NRRL 5167 | Cultured | [12] |
| Mangrovamide A | *Penicillium* sp. | Mangrove sediment sample, South China Sea, China | [13] |
| Mangrovamide B | *Penicillium* sp. | Mangrove sediment sample, South China Sea, China | [13] |
| Mangrovamide C | *Penicillium* sp. | Mangrove sediment sample, South China Sea, China | [13] |
| Penioxalamine A | *Penicillium oxalicum* TW01-1 | Soil, Xitou mountain, Taiwan | [14] |
| Marcfortine A = PNU-94119 | *Penicilliurn roqueforti,* | Cultured | [15] |
| 14-Hydroxymarcfortine A = PNU-99437 | *-* | - | [3] |
| 14-Hydroxymethylmarcfortine A = PNU-99809 | *-* | - | [3] |
| Marcfortine B | *Penicilliurn roqueforti,* | Cultured | [16] |
| Marcfortine C | *Penicilliurn roqueforti,* | Cultured | [16] |
| Malbrancheamide | *Malbranchea aurantiaca* | Bat guano, Juxtlahuaca cave, Ramal del Infierno, Guerrero, Mexico | [17] |
|  | *Malbranchea aurantiaca* | Cultured | [18] |
| Premalbrancheamide | *Malbranchea aurantiaca* | Cultured | [18] |
| Malbrancheamide B | *Malbranchea aurantiaca* | Bat guano, Juxtlahuaca cave, Ramal del Infierno, Guerrero, Mexico | [17] |
|  | *Malbranchea aurantiaca* | Cultured | [18] |
| Isomalbrancheamide B | *Malbranchea aurantiaca* | Cultured | [18] |
| Malbrancheamide C | *Malbranchea graminicola* | Cultured | [18] |
| Isomalbrancheamide C | *Malbranchea graminicola* | Cultured | [18] |
| Malbrancheamide D | *Malbranchea graminicola* | Cultured | [18] |
| Isomalbrancheamide D | *Malbranchea graminicola* | Cultured | [18] |
| Aspergamide A | *-* | - | [19] |
| Aspergamide B | *-* | - | [19] |
| Avrainvillamide = CJ-17,665 | *Aspergillus ochraceus* | Soil sample, Venezuela | [19,20] |
| Stephacidin A | *Aspergillus ochraceus* WC76466 | Cultured | [21] |
| Stephacidin B | *Aspergillus ochraceus* WC76466 | Cultured | [21] |
| Waikialoid A | *Aspergillus* sp. | Soil sample, Waikiki Beach, Honolulu, Hawaii | [22] |
| Waikialoid B | *Aspergillus* sp. | Soil sample, Waikiki Beach, Honolulu, Hawaii | [22] |
| Chrysogenamide | *Penicillium chrysogenum* No. 005 | Root, *Cistanche deserticola*, northwest China | [23] |

*Both compounds have same names but different

**References**

1. Yamazaki M, Okuyama E, Kobayashi M, Inoue H. The structure of paraherquamide, a toxic metabolite from *Penicillium paraherquei*. Tetrahedron Lett. 1981;22: 135-136.

2. Liesch JM, Wichmann CF. Novel antinematodal and antiparasitic agents from *Penicillium charlesh* II. Structure determination of paraherquamides B, C, D, E, F, and G. J Antibiot. 1990;43: 1380-1386.

3. Zinser EW, Wolf ML, Alexander‐Bowman SJ, Thomas EM, Davis JP, Groppi VE, et al. Anthelmintic paraherquamides are cholinergic antagonists in gastrointestinal nematodes and mammals. J Vet Pharmacol Ther. 2002;25: 241-250.

4. Zheng Y, Shen N, Liang Z, Shen L, Chen M, Wang C. Paraherquamide J, a new prenylated indole alkaloid from the marine-derived fungus *Penicillium janthinellum* HK1-6. Nat Prod Res. 2020;34: 378-384.

5. Ondeyka JG, Goegelman RT, Schaeffer JM, Kelemen L, Zitano L. Novel antinematodal and antiparasitic agents from *Penicillium charlesii* I. Fermentation, isolation and biological activity. J Antibiot. 1990;43: 1375-1379.

6. Wu J, Wang F, He L, Zhou S, Wang S, Jia J, et al. Aculeaquamide A, cytotoxic paraherquamide from the marine fungus *Aspergillus aculeatinus* WHUF0198. Nat Prod Res. 2022;36: 4382-4387.

7. Blanchflower SE, Banks RM, Everett JR, Manger BR, Reading C. New paraherquamide antibiotics with anthelmintic activity. J Antibiot. 1991;44: 492-497.

8. Fraley AE, Caddell Haatveit K, Ye Y, Kelly SP, Newmister SA, Yu F, et al. Molecular basis for spirocycle formation in the paraherquamide biosynthetic pathway. J Am Chem Soc. 2020;142: 2244-2252.

9. Lopez-Gresa MP, Gonzalez MC, Ciavatta L, Ayala I, Moya P, Primo J. Insecticidal activity of paraherquamides, including paraherquamide H and paraherquamide I, two new alkaloids isolated from *Penicillium cluniae*. J Agric Food Chem. 2006;54: 2921-2925.

10. Blanchflower SE, Banks RM, Everett JR, Reading C. Further novel metabolites of the paraherquamide family. J Antibiot. 1993;46: 1355-1363.

11. Banks RM, Blanchflower SE, Everett JR, Manger BR, Reading C. Novel anthelmintic metabolites from an *Aspergillus* species; the aspergillimides. J Antibiot. 1997;50: 840-846.

12. Whyte AC, Gloer JB, Wicklow DT, Dowd PF. Sclerotiamide: a new member of the paraherquamide class with potent antiinsectan activity from the sclerotia of *Aspergillus sclerotiorum*. J Nat Prod. 1996;59: 1093-1095.

13. Yang B, Dong J, Lin X, Zhou X, Zhang Y, Liu Y. New prenylated indole alkaloids from fungus *Penicillium* sp. derived of mangrove soil sample. Tetrahedron. 2014;70: 3859-3863.

14. Hu X, Bian X, Wu X, Li J, Hua H, Pei Y, et al. Penioxalamine A, a novel prenylated spiro-oxindole alkaloid from *Penicillium oxalicum* TW01-1. Tetrahedron Lett. 2014;55: 3864-3867.

15. Polonsky J, Merrien M, Prangé T, Pascard C, Moreau S. Isolation and structure (X-ray analysis) of marcfortine A, a new alkaloid from Penicillium roqueforti. J Am Chem Soc, Chem Commun. 1980: 601-602.

16. Prange T, Billion M, Vuilhorgne M, Pascard C, Polonsky J, Moreau S. Structures of marcfortine B and C (X-ray analysis), alkaloids from *Penicillium roqueforti*. Tetrahedron Lett. 1981;22: 1977-1980.

17. Figueroa M, González MDC, Mata R. Malbrancheamide B, a novel compound from the fungus *Malbranchea aurantiaca*. Nat Prod Res. 2008;22: 709-714.

18. Fraley AE, Garcia-Borràs M, Tripathi A, Khare D, Mercado-Marin EV, Tran H, et al. Function and structure of MalA/MalA′, iterative halogenases for late-stage C–H functionalization of indole alkaloids. J Am Chem Soc. 2017;139: 12060-12068.

19. Williams RM, Cox RJ. Paraherquamides, brevianamides, and asperparalines: laboratory synthesis and biosynthesis. An interim report. Acc Chem Res. 2003;36: 127-139.

20. Sugie Y, Hirai H, Inagaki T, Ishiguro M, Kim Y, Kojima Y, et al. A new antibiotic CJ-17, 665 from *Aspergillus ochraceus.* J Antibiot. 2001;54: 911-916.

21. von Nussbaum F. Stephacidin B-a new stage of complexity within prenylated indole alkaloids from fungi. Angewandte Chemie International Edition. 2003;42: 3068-3071.

22. Wang X, You J, King JB, Powell DR, Cichewicz RH. Waikialoid A suppresses hyphal morphogenesis and inhibits biofilm development in pathogenic *Candida albicans*. J Nat Prod. 2012;75: 707-715.

23. Lin Z, Wen J, Zhu T, Fang Y, Gu Q, Zhu W. Chrysogenamide A from an endophytic fungus associated with *Cistanche deserticola* and its neuroprotective effect on SH-SY5Y cells. J Antibiot. 2008;61: 81-85.
